# *In Vitro* Cellular Strain Models of Tendon Biology and Tenogenic Differentiation

**DOI:** 10.3389/fbioe.2022.826748

**Published:** 2022-02-15

**Authors:** Shannon Y. Wu, Won Kim, Thomas J. Kremen

**Affiliations:** ^1^ David Geffen School of Medicine at UCLA, Los Angeles, CA, United States; ^2^ Department of Rehabilitation Medicine, Asan Medical Center, University of Ulsan College of Medicine, Seoul, South Korea; ^3^ Department of Orthopaedic Surgery, David Geffen School of Medicine at UCLA, Los Angeles, CA, United States

**Keywords:** mechanotransduction, tendon, tenogenic differentiation, bioreactor, 3D culture, model

## Abstract

Research has shown that the surrounding biomechanical environment plays a significant role in the development, differentiation, repair, and degradation of tendon, but the interactions between tendon cells and the forces they experience are complex. *In vitro* mechanical stimulation models attempt to understand the effects of mechanical load on tendon and connective tissue progenitor cells. This article reviews multiple mechanical stimulation models used to study tendon mechanobiology and provides an overview of the current progress in modelling the complex native biomechanical environment of tendon. Though great strides have been made in advancing the understanding of the role of mechanical stimulation in tendon development, damage, and repair, there exists no ideal *in vitro* model. Further comparative studies and careful consideration of loading parameters, cell populations, and biochemical additives may further offer new insight into an ideal model for the support of tendon regeneration studies.

## Introduction

Tendons are connective tissues responsible for transmitting the force generated by muscles to bones. Although their structure and composition allow them to tolerate repetitive forces during daily activities, excessive or abnormal applications of force can cause tendon damage or rupture. Tendon repair can be difficult due to the hypocellular and avascular nature of tendon tissue. Emerging tissue engineering strategies continue to be developed with the goal of augmenting current treatment approaches as well as the development of novel therapies for the treatment of tendon injuries. Although exposure to excessive force contributes to tendon injury, mechanical loading of tendon tissue also plays an important role in appropriate tendon healing. As a result, *in vitro* mechanical stimulation studies attempt to understand the effects of mechanical load on tendon and connective tissue progenitor cells that have the potential to differentiate into tendon cells. These models have allowed researchers to observe the effects of mechanical loading on these cells with regard to tendon gene expression, cell morphology, protein production as well as more gross tissue properties including cellular alignment and tensile strength. This review aims to summarize and compare the different cellular mechanical loading models used in the study of tendon mechanobiology and highlight how the designs of these models have evolved over time in the effort to recapitulate the complexity of forces exerted upon tendon tissues *in vivo*. First, we contextualize our discussion with an introduction to current understanding of native tendon structure and biomechanics. We then discuss how explanted tendon, 2D, and 3D models have contributed to our understanding of the effects of mechanical stimulation in the effort to recapitulate the native tendon architecture in the *in vitro* setting. We conclude by identifying existing gaps in knowledge and possible directions for future work within the field of *in vitro* mechanical loading research.

## Structure and Biomechanics of Tendon

### Structure of Tendon

Tendons are hypocellular structures that largely consist of water and extracellular matrix (ECM), with water making up 55–70% of the tissue mass.(Jaiswal et al., 2020). ECM is predominantly composed of various types of collagen protein molecules. Type I collagen accounts for 60–85% of the total dry weight of tendon tissue (Docheva et al., 2015), whereas type III collagen accounts for 1–5% of the total collagen content.(Liu et al., 1995; Lim et al., 2019). There are also small amounts of other collagen proteins, such as type II, IV, V, XII and XIV collagen (Wang, 2006; Wang et al., 2018a). Type III collagen plays an important role in regulating the size of type I collagen fibrils, while type V collagen, found in the center of fibrils, is thought to act as the template for fibrillogenesis. (Thorpe and Screen, 2016).

Tendon’s mechanical properties can be attributed to its hierarchical structure of collagen fibrils (Trelstad and Hayashi, 1979; Wang, 2006). At the molecular level, collagen’s triple helix structure allows tendon tissues to have sufficient mechanical strength. (Fratzl et al., 1998). The intermolecular sliding of collagen within tendon facilitates deformation in a linear fashion with application of strain (Kelc et al., 2013). Collagen fibrillogenesis, or the formation of fibrils from the collagen molecules, is an important multi-step process that determines the functionality of the overall tendon. There has been debate on how this process unfolds as tendons mature. Some believe that collagen molecules assemble to form fibril intermediates, which then assemble end-to-end to create longer and mechanically functional fibrils. These longer fibrils then interact laterally, forming fibrils of a larger diameter. In contrast, Kalson et al. have suggested that end-to-end and lateral fusion events do not contribute to fibril growth, as serial microscopy has revealed few fusion events during development, and the number of fibrils does not decrease as expected with fusion. (Kalson et al., 2015). Rather, collagen molecules may accrue on fibril ends, growing in a binding site- and structure-specific mechanism as described by the surface-nucleation-and-propagation (SNP) model. (Holmes et al., 1998). Collagen fibril bundles form fibers, which in turn form the fascicles of tendon tissues. These structures are arranged parallel to the long axis of tendon tissues, which give tendon tissue its hallmark tensile strength (Wang, 2006). Some authors have posited that fascicles are further organized into a spiral formation (Kannus, 2000; Ottani et al., 2002). While some argue that such a structure would make transference of force to bone less efficient, this architecture may be better suited to resisting flexion, compression, and multidirectional forces.

Non-collagenous proteins found within tendon extracellular matrix (ECM) consist of a variety of proteoglycan (PG) molecules (e.g. decorin, fibromodulin, lumican, and fibromodulin), glycoproteins (e.g. tenascin C, fibronectin, lubricin, and laminin) and glycosaminoglycans (Humphrey et al., 2014). The hydrophilic nature of these proteins help the structure to retain high water content, which provides some resistance to physiologic compression forces and inter-fascicular sliding (Jozsa et al., 1989; Kirkendall and Garrett, 1997; Martin et al., 2003; Vogel, 2004; Kohrs et al., 2011; Docheva et al., 2015; Wunderli et al., 2020). In addition, proteoglycans play important roles in collagen fibrillogenesis. Lumican, fibromodulin, decorin, and biglycan all play a role in stabilizing fibril formation and maturation, as knockout mouse models demonstrate that elimination of these proteoglycans result in weaker tendons and increased fibrosis (Robbins et al., 1997; Ezura et al., 2000; Zhang et al., 2009). Proteoglycans may also play a role in attenuating strain experienced at the cellular level. Studies have found that the levels of strain transferred to cells in synthetic scaffolds differ from native levels, as significant strain attenuation occurs from the tissue level to the cell level (Cheng and Screen, 2007). At times, levels of strain estimated on synthetic scaffolds can overestimate native tissue strain by 20–60% (Han et al., 2013). The authors further found that cells in the PG-rich portions of the sample experienced less strain than those in more fibrous areas, suggesting that PGs may affect the local mechanical response, possibly through support of collagen fibrillogenesis or facilitation of resistance to compression or sliding forces.

### Biomechanical Properties of Tendon

Tendon tissues have specialized mechanical properties and have features of linear elastic material models as well as viscoelastic material models. In fact, mathematical methods termed quasi-linear viscoelasticity models have been used to model tendon tissue’s mechanical behavior (Woo et al., 2000).

The stress-strain curves of tendon tissues are of particular interest because they demonstrate their intrinsic tensile strength during pull-to-failure testing. When using linear elastic material models to describe tendon mechanical properties, the stress-strain curves have three regions, including the toe region, the linear region, and the yield or failure region. (Hooley et al., 1980; Lieber et al., 1991). The toe region corresponds with stretching of crimped fiber bundles, which continues until 2% of strain (Wang, 2006). Within the toe region, the crimp pattern of tendon fibers is stretched; it is recovered when the stress disappears (Lim et al., 2019). Within the linear region (2–6% strain), the slope of the stress-strain curve appears to be constant as it is the inflection point where the curve transitions from strain stiffening to strain softening. This region is also the point at which the tangent modulus reaches its maximum value. Studies have found that over 4% strain can cause microscopic damage during the sliding of collagen fibrils (Thompson et al., 2017). The failure region of the stress-strain curve demonstrates macroscopic tears associated with strain values over 8–10% (Butler et al., 2000; Thompson et al., 2017).

Viscoelastic materials demonstrate different mechanical properties depending on the rate of loading (Woo et al., 2000; Robi et al., 2013). One characteristic of viscoelastic materials such as tendons is creep, the deformation of a material in response to constant load (Jaiswal et al., 2020). *Creep test* or “fatigue” test is used to demonstrate response of tendon to continuous or repetitive loading (Wang et al., 2018a). Creep testing helps explain the relation between time and strain under constant load (Wren et al., 2003). Upon exposure to initial load, the deformation of tendon tissue is temporary, but as the load is sustained, damage accumulates, and the subsequent strength and stiffness of the damaged tendon tissue decreases (Wang et al., 1995). It is also worth noting that the mechanical properties of different tendons varies by tendon anatomic location and function (Wang et al., 1995; Maganaris and Paul, 1999; Shani et al., 2016).

Mechanical loading, even within the range of typical of daily activities, can lead to tendon damage. *In vitro* mechanical loading studies have reported that mechanical degradation can occur within 24 h of exposure to very low levels of repetitive stress, at a level less than 5% of the ultimate tensile strength of the tissue (Parent et al., 2011). However, this observed *in vitro* degradation would likely be different *in vivo* due to the repair mechanisms of host tendon tissues and the associated supporting cells (e.g. resident connective tissue progenitor cells and immune system cells). Based on results of *in vitro* studies, Wang et al. have suggested that physiologic loading triggers tendon repair, such that the repair counteracts microscopic damage (Wang et al., 2003; Wang et al., 2015). In particular, the therapeutic effects of eccentric loading have been extensively studied in the context of treating Achilles tendinopathy (Fahlström et al., 2003). While human studies reveal that eccentric loading tends to increase the maximum force and the maximum number of repetitions tendons can sustain (Malliaras et al., 2013), the mechanism by which this form of loading has been unclear (O’Neill et al., 2015). A possible explanation can be attributed to complex muscle-tendon interactions, by which the muscle shields tendon from stress by storing energy or increasing tendon stiffness, thereby reducing damage (Sugisaki et al., 2011; Roberts and Konow, 2013; O’Neill et al., 2015). Neuromodulatory effects that facilitate adaptive or protective motor patterns may also explain therapeutic effects of eccentric loading (Rees et al., 2008; Rio et al., 2016). These interactions of the tendon with the surrounding tissue have only been captured in live patients or animal models. Therefore, effects of repair may be more significant *in vivo,* in the presence of supporting cells and chemical signals which may more readily trigger healing. This may explain the discrepancy between ruptures observed with creep testing of explanted tendon tissues and the long-term functionality of tendon tissues *in vivo* (Wang et al., 2018a).

However, some degree of mechanical stimulation is required to maintain normal tendon structural and mechanical properties. Live studies have revealed that stress shielding can cause deterioration of tendon tissue. Early studies assessing the effects of completely releasing stress in various live animal models revealed that collagen bundles were less aligned, and fibrils tended to be smaller in diameter (Muellner et al., 2001; Majima et al., 2003). Stress shielding also decreased the mechanical strength and strain-at-failure of collagen fascicles (Yamamoto et al., 1999). These studies reaffirm that some level of mechanical stimulation may be essential to optimal maintenance and repair of tendon structure *in vivo*; this suggests that complete deprivation of stress in the *in vitro* setting may cause tenocytes and tendon explants to lose tendon-like properties as well.

While tensile loads are the main mechanical stress to which tendon are exposed, other forces may contribute to physiologic or pathologic responses to applied forces. Compressive forces have been suggested as a cause of tendon damage.(Almekinders et al., 2003; Hamilton and Purdam, 2004). In addition, interfibrillar shear stress and fluid shear stress also have been described in tendon tissue (Arnoczky et al., 2002a; Lavagnino et al., 2008; Szczesny and Elliott, 2014; Szczesny et al., 2015; Lee and Elliott, 2019). Low levels of fluid shear stress resulted in lower levels of collagenase mRNA inhibition, indicating that this form of mechanical stimulation plays a significant role in the preservation of collagen matrix integrity. (Lavagnino et al., 2008). Sliding and rotational behaviors in the fibers of tendon samples have also been observed; studies investigating the transference of applied force to cells also found that applied levels of strain were significantly attenuated at the fiber level, suggesting that these sliding forces may play a role in attenuating forces experienced by tendon. (Cheng and Screen, 2007; Lee and Elliott, 2019). In energy-storing tendons such as flexor tendons, fascicular sliding and interfascicular matrix (IFM) elasticity facilitated these tendons’ ability to resist and recover from higher loads (Thorpe et al., 2015). Thus, sliding forces may play an important role in tendon behavior at multiple levels of organization and moderate the level of strain exerted at the cellular level.

## Tendon Cell Response to Mechanical load

Mechanical stimulation is essential for tendon development, maturation, and repair, but the underlying mechanisms of tendon mechanoresponse elements remain poorly understood. Tendon cell and progenitor cell biology are known to be influenced by mechanical factors, (Engler et al., 2006), the response to which is mediated by the ECM itself, mechanosensors, and effector molecules (Banes et al., 1999). Matrix stiffness, discussed later in 2D model studies, has been shown to strongly influence stem cell differentiation, as demonstrated by changes in cell morphology as well as gene expression and protein production. In addition to general mechanoresponse pathways *via* membrane proteins and signalling cascades, studies have added to a growing body of knowledge of tendon-specific pathways.

Tendon-specific pathways for mechanoresponse have recently been described. (Fang et al., 2020; Passini et al., 2021). At the enthesis, cilia transduce biophysical activity *via* hedgehog (Hh) signaling to facilitate adaptation to load in an inverse relationship (Fang et al., 2020). Mechanical stimulation caused cilia disassembly, a process disrupted with the loss of Hh signaling. Deletion of ciliary genes in mice resulted in weakened entheses and decreased enthesis mineralization, suggesting that cilia may play a role in the enthesis mechanoresponse mechanisms and may also be influenced to affect pathology resulting from overloading. In late 2021, Passini et al. published their findings that intracellular calcium signaling occurs among explanted rat tendon fascicles in response to stretch (Passini et al., 2021). At low strain rates (0.1% strain/second), tenocytes in their *ex vivo* model exhibited multiple intracellular Ca2+ signals over a longer duration, while at high strain rates (1.0% strain/second), tenocytes displayed a single shorter duration intracellular Ca2+ response. They also noted that, as strain rate increased, higher tissue stretch was required to elicit a response among 50% of the cells in their model. Passini et al. also demonstrated that tenocytes, which reside between collagen fibers and are thus exposed to mechanical shear during tissue stretching, are able to detected these shear-stress forces *via* the mechanosensitive ion channel PIEZO1. The authors proposed that the Ca2+ signals observed in their experiments may in turn activate enzymatic cross linking of collagen, contributing to increased tendon stiffness and strength and, ultimately, affecting physical performance This pathway suggests a mechanism by which tendon mechanoresponses to shearing and stretching forces are optimized in order to maintain tendon morphology and biomechanical properties.

More generally, cells, including those in tendon, may detect mechanical stimulation *via* transmembrane proteins that are integrated with cytoskeletal elements and non-receptor signaling molecules. Though studied in other mesenchymal cell populations and not unique to tendon, these pathways nevertheless play an important role in guiding the cellular response to mechanical stimulation. These mechanosensing cellular structures and surface molecules include integrins, cadherins, and connexins. (Chrzanowska-Wodnicka and Burridge, 1996; Lavagnino et al., 2011; Leckband et al., 2011; Schiele et al., 2013). For example, forces on ECM-integrin-cytoskeletal linkages may initiate signal pathways by altering protein folding or binding affinity. For example, studies have shown that stretching forces can expose binding sites on fibronectin promoting self-assembly into fibrils. (Morla and Ruoslahti, 1992; Zhong et al., 1998). Cytoskeleton deformation by mechanical stimulation can also alter transcriptional activities within the cell.(Wang et al., 2009; Tajik et al., 2016). Integrins can sense forces to which a cell is exposed and then propagate that forces to the actin cytoskeleton, nuclear laminins and eventually chromatin, which causes stretching and access to target genes for transcription.(Tajik et al., 2016). Pathways for stem cell differentiation may also be activated in this manner where actin filaments rapidly polymerize and depolymerize in response to mechanical loading, which in turn leads to translocation of MKL1 (megakaryoblastic leukemia 1) into the nucleus. MKL1 protein is shown to interact with the beta catenin (Wnt) and Smads (TGF-beta) pathways, which can further guide cellular differentiation, migration, and cell cycle regulation.(Scharenberg et al., 2014). Increased MKL1-actin activity reduces chromatin access, leading to reduction of cell pluripotency.(Hu et al., 2019).

The YAP/TAZ pathway may be another non-specific pathway that may be activated in response to tendon mechanical stimulation. YAP (Yes-associated protein) and TAZ (transcriptional co-activator with PDZ-binding motif) effector proteins in the Hippo pathway have been found to be influenced by ECM stiffness and cytoskeleton organization. (Dupont et al., 2011). YAP/TAZ play a key role in regulating the quiescence, proliferation, and differentiation of adult progenitor cells in multiple tissues including tendon. (Heng et al., 2020). In an environment with a stiff ECM or cellular attachment substrate, increased integrin clustering and focal adhesion formation increases F-actin polymerization, which in turn induces cell spreading. This then promotes translocation of YAP/TAZ into the nucleus, which consequently increases osteogenesis *via* upregulation of *RUNX2* and downregulation of *PPAR-γ*. On soft substrates, cells retain a rounder shape due to less focal adhesion formation and actin polymerization, which sequesters YAP/TAZ to the cytosol. This sequestration consequently downregulates *RUNX2* (runt-related transcription factor 2) and upregulates *PPAR-γ* (peroxisome proliferator-activated receptor gamma), driving adipogenesis. In particular, TAZ activation has been consistently reported to play a role in osteogenesis, while the role of YAP remains controversial.(Heng et al., 2020). However, Yeung et al. have reported upregulation of YAP/TAZ target genes during tendon development, possibly reflecting the concurrent deposition of ECM components. (Yeung et al., 2015).

## *In vitro* Tendon Cell Loading Models

Numerous loading models have been developed to study the mechanobiology of tendon. The scope of this review is limited to *in vitro* loading models, which can be grossly separated into explant, 2-dimensional (2D), or 3-dimensional (3D) loading models. The ideal *in vitro* model and loading conditions for tendon tissue engineering recapitulates the various mechanical forces transmitted through the ECM *in vivo* to native tendon cells while also maintaining cell viability. Desirable characteristics of optimal tendon tissue models include the generation of a well-aligned cellular morphology, the differentiation of progenitor cells towards a tendon-like phenotype, and the production of ECM proteins commonly observed among tendon tissues.(Butler et al., 2000; Brennan et al., 2018). These models must also have the capacity to tolerate the application of physiologically-relevant forces. Tendon tissues *in vivo* are usually subjected to uniaxial loading at a level of approximately 4–6% strain, (Screen et al., 2004), though higher and lower amounts of uniaxial loads may be utilized to replicate pathologic states. Tendon-derived stem cells (TDSCs) are endogenous progenitor cells within tendon tissues and have been isolated from both animal and human sources (Rui et al., 2010; Lui and Chan, 2011). While TDSCs may be the most relevant MSC population for tendon regeneration research, studies have found that bone marrow or adipose derived MSCs are robust alternatives.(Yin et al., 2016; Youngstrom et al., 2016). The following two sections summarize the main categories of *in vitro* tenogenic mechanical loading models as well as key principles and findings associated with the use of these models.

### *Ex vivo* Tendon Loading Models

Tendon explants as an *ex vivo* loading model provide useful insight into the mechanical behavior of isolated tendons. Native cell-cell and cell-matrix interactions are preserved and may thus be investigated, providing information not otherwise available through 2D and 3D models. 2D and 3D models also currently lack the heterogeneity in cell population found in native tendon.(Viganò et al., 2017). These mechanical studies produced some of the earliest knowledge about cell-cell interactions, intrinsic tendon mechanical properties, cell viability and growth, and gene expression.

Strain levels used in *ex vivo* studies range from 1 to 9%, though strains of 20–30% have been applied to model tendon injury.(Scott et al., 2005; Legerlotz et al., 2013). A general summary of the outcomes of different levels of applied strain is given in Table 1, and loading parameters used in individual studies are given in Supplementary Table S1. Cyclic strain from 1 to 3% on tendon fascicles and whole-tendon samples have been sufficient to preserve tenocyte-like morphology and tendon specific gene expression, (Maeda et al., 2009; Wang et al., 2013; Wunderli et al., 2018; Tohidnezhad et al., 2020), but matrix deterioration and reduced maximum stress and energy density were also noted at these levels of strain.(Devkota et al., 2007; Wang et al., 2013). Physiologic loads of 5–6% also yielded increased collagen production and can rescue unloaded tendon samples from pathologic changes.(Wang et al., 2015). Overloading of explants revealed degenerative changes in ECM and increased apoptosis and inflammatory markers.(Legerlotz et al., 2013; Wang et al., 2013). Failure-loaded samples were notable for collagen fiber kinking and denaturation.(Szczesny et al., 2018). Interestingly, the lack of change in tissue modulus suggests that changes in tissue architecture initiate the first changes in tenocyte phenotype and cell viability during injury. This hypothesis has been supported by Freedman et al.‘s work which found that macroscale damage and matrix disorganization caused by fatigue loading propagates down to cellular level; damaged matrix may transmit forces differently than uninjured matrix, thus spurring differential cellular responses.(Freedman et al., 2018). In summary, low to physiologic loading preserved and sometimes rescued tenogenic genes and ECM production in explants, while high strains more consistently caused structural damage, which consequently affects cellular responses due to changes in detected mechanical strain.

**TABLE 1:** Overview of outcomes of applied mechanical strain to tendon explants and fascicles.

Level of strain	General outcomes
1–4%	Preservation of tendon and matrix genes including *SCX, TNMD*, *Ctgf* Wunderli et al. (2018), Tohidnezhad et al. (2020)
	Downregulation of decorin Maeda et al. (2009)
	Enhanced collagen production Maeda et al. (2007)
	Matrix deterioration at 3% strain Wang et al. (2013)
	Reduced maximum stress and energy density, increased cell death and collagenase activity; increased glycosaminoglycan content, increased PGE2 Devkota et al. (2007)
	Increased *MMP3*, *MMP13*, *MMP9* (Maeda et al. (2009); Tohidnezhad et al. (2020)
5–8%	Retention of structural integrity, cellular function Wang et al. (2013)
	Increased collagen production Wang et al. (2015)
	Rescue of unloaded tendon samples from pathologic changes Wang et al. (2015)
	Increased collagen synthesis Screen et al. (2005)
9%+	Increased *COL1A1*, *IL-6* Legerlotz et al. (2013)
	Massive collagen bundle rupture, fiber kinking and denaturationWang et al.(2013);Szczesny et al. (2018)
	Induced stretch overload injury with increased apoptosis and abnormal nuclear morphologyScott et al. (2005)

Because researchers can avoid the challenges in finetuning scaffold properties in *ex vivo* studies, important findings have been produced from comparing different loads. Interestingly, *ex vivo* studies of tendon explants from various species have revealed that healthy tendons can demonstrate auxetic behavior when placed under biaxial strain—that is, the tendons become thicker, rather than thinner, when stretched.(Gatt et al., 2015). Furthermore, this auxetic behavior was highly influenced by tendon microstructure, which may be compromised by damage due to excessive loads or altered tendon biology. A 3D hydrogel scaffold with auxetic properties was developed to aid in future tendon-to-muscle regeneration (Warner et al., 2017), but beyond cell viability studies, there remains little information about the effects of this scaffold with regard to guiding tenogenic differentiation among mesenchymal progenitor cells.

### Two-Dimensional (2D) Loading Models

Two-dimensional loading models study the effects of mechanical stimulation, with or without additional dynamic loading, on cells grown in monolayer. In the case of dynamic loading, cells adhere to a flexible material to which mechanical strain can be applied. The stiffness of the substrate on which cells are plated has also been shown to affect tenogenic gene expression among progenitor cells.(Sharma and Snedeker, 2010). Most other models, including commercially available units such as those made by Flexcell^®^, use the cyclical application of negative pressure (via a vacuum device) to homogenously apply mechanical strain to the flexible material and the attached cells. This provides customizable loading conditions for experiments. 2D models mainly investigate the effects of deforming cellular cytoskeletons and nuclei, providing an approach to evaluate certain cell differentiation and migration pathways. Depending on the cell population seeded, the aim of the study may be to study mechanical stimulation’s effects on the preservation of tendon-specific genes or morphology in tendon cells or the differentiation of progenitor cells towards a tenogenic fate. However, 2D models lack a substantial ECM component and thus are not well-suited to replicate cell-ECM interactions, which is a major limitation of these techniques.

### 2D Mechanical Stimulation

Static mechanical stimulation of cells in monolayer may be achieved via specialized plates with defined mechanical properties. Culture plates composed of materials with different degrees of mechanical stiffness, functionalized with type I collagen or fibronectin cellular adhesion molecules, have been reported to guide the fate of bone marrow stem cells (BMSCs) towards tendon or bone phenotypes. Snedeker et al. plated BMSCs onto gel strips with stiffnesses ranging from 10 kPa to 100 kPa and found that increased *RUNX2* expression was associated with increased stiffness of a fibronectin-coated substrate. (Sharma and Snedeker, 2010). Gene expression of scleraxis (*SCX*), tenascin-C (*TNC*), tenomodulin (*TNMD*), and collagen III (*COL3*) have been noted to be increased among cells plated on collagen substrates within a more narrow range of stiffness (approximately 30–50 kPa), even in the absence of additional dynamic mechanical load.(Sharma and Snedeker, 2010, 2012). The authors hypothesize that since *SCX* expression can be affected by MAP kinase activity, and MAP kinase can be influenced by mechanical stimulation, it is possible that stiffer substrates stimulate a level of MAP kinase activity that does not permit tenogenesis, while softer substrates may provide insufficient mechanical stimulation. Islam et al. found a similar upregulation of tenogenic markers in mesenchymal stem cells (MSCs) plated on type-1 collagen sheets of varying stiffness. Higher levels of *SCX* were seen in cells on sheets with a modulus of 100 MPa compared to 0.1 MPa.(Islam et al., 2017). In addition, architectural organization of the substrate influenced tenogenesis and ECM formation. 10 MPa collagen sheets with highly aligned fibers demonstrated comparable increases in type-III collagen gene expression and cartilage oligomeric matrix protein (COMP, a protein involved in tendon load resistance) (Smith et al., 1997) as an unaligned 100 MPa sheet.(Islam et al., 2017). Finally, *RUNX2* gene expression was significantly increased after 21 days in cells seeded on the 100 MPa sheet, suggesting that the same conditions for stiffness that promote tenogenesis also promote osteogenesis over time.(Islam et al., 2017). Researchers should be aware of the time-dependent and substrate-dependent nature of tenogenic differentiation to achieve optimal conditions for tendon or tendon-like tissue formation.

### 2D Dynamic Mechanical Loading

Studies of biaxial or uniaxial dynamic loading have also been studied on 2D models. Biaxial loading generates a multidirectional strain (e.g. radial strain exerted on monolayer of cells adherent to a flexible substrate). Although this strain model does not typically replicate typical physiologic tendon loading, cells undergoing cyclic biaxial strain have shown increased proliferation, collagen production, and cell morphology changes similar to those observed among tenocytes.(Zeichen et al., 2000; Goodman et al., 2004; Deniz et al., 2020). Uniaxial strain more closely replicates loads experienced by native tendon cells, and thus the majority of mechanical loading research has focused on the effects of uniaxial strain on tendon and stem cells. Similar to cyclic biaxial strain, cyclic uniaxial strain has also increased collagen I production, cell proliferation, and cell alignment.(Yang et al., 2004; Riboh et al., 2008). As with static loading, duration of application of strain appear to influence cell behavior; for example, Jun N-terminal kinase 1 (JNK) pathway-mediated apoptosis was noted to be elevated over brief applications of cyclic uniaxial strain, however, after long-term exposure to strain, no differences in levels of tenocyte apoptosis were found. This implies that tenocytes may have the capacity to develop stress tolerance during prolonged periods of mechanical loading and this phenomenon may be evolutionarily and functionally beneficial *in vivo*.(Skutek et al., 2003). Although few studies have directly compared the effects of biaxial versus uniaxial strain on cell behavior, Wall et al*.* found that biaxial substrate strain induces lower levels of cellular strain compared to uniaxial substrate strain.(Wall et al., 2007). Wang et al. found that while uniaxial strain consistently increased expression of *SCX*, Mohawk (*MKX*), and *TNMD*, biaxial loading actually inhibited expression of those markers.(Wang et al., 2018b).

Customizable mechanical loads have afforded observation of the effects of different levels of strain on tenogenic differentiation and morphology. Though low and high strain generally model tendon behavior seen in pathological conditions, results from these studies have been varied (Tables 2, 3). Loading parameters of studies discussed are included in Supplementary Table S2. Low magnitude loads of 2–4% have increased early gene expression of inflammatory factors and ECM degradation enzymes, (Tsuzaki et al., 2003), and tendon-derived stem cells under low strain have also demonstrated upregulated osteogenic and chondrogenic markers, including bone morphogenic protein (*BMP-2*) expression. (Chen et al., 2008; Rui et al., 2011). Conversely, low strain loading also increased tenogenic gene expression, including *MKX*, *TNMD*, and collagen, type 1, alpha 1 and 2 (*COL1A1*, *COL1A2*)*.*(Zhang and Wang, 2010; Kayama et al., 2016). Of note, Kayama et al. did not find an increase in *SCX* expression with 2% cell strain.(Kayama et al., 2016). It is possible that 2% strain, or the duration of 2% strain used in the study, is insufficient to increase expression of that specific marker.

**TABLE 2:** Overview of outcomes of applied mechanical strain to 2D loading studies using human cells (stem cells and tendon fibroblasts).

Level of strain	General outcomes
1–4%	Increased collagen type I, TGF- β Yang et al. (2004)
	Decreased COX-2, MMP-1, PGE2 gene expression Yang et al. (2005)
	Increased COX-2, IL-1 β, MMP-3 gene expression Tsuzaki et al. (2003), Wang et al. (2003)
	Transient increases in *ALP*, *RUNX2*, *OCN* gene expression Chen et al. (2008), Nam et al. (2019)
5–8%	Increased SCX*, TNMD*, *TNC* gene expression Nam et al. (2019)
	Increased TGF- β Yang et al. (2004)
	Increased collagen I, collagen III, fibronectin, N-cadherin Yang et al.(2004); Nam et al. (2019)
	Increased COX-2, MMP-1, PGE2 expression Yang et al. (2005)
	Increased JNK activation and apoptosis of tendon fibroblasts Skutek et al. (2003)
9%+	Increased *TNC*, *SCX*, *TNMD* gene expression in BMSCs Chen et al. (2008); Nam et al. (2019)
	Increased collagen type I, collagen type III, fibronectin, N-cadherin Chen et al. (2008); Nam et al. (2019)
	Increased PGE2, COX-1, COX-2 Wang et al. (2003)
	Transiently increased *ANGPTL4,* FGF-2*, COX-2, SPHK1*, TGF-alpha*, VEGF-A* and *VEGF-C* Mousavizadeh et al. (2013)

**TABLE 3:** Overview of outcomes of applied mechanical strain to 2D loading studies using animal cells (stem cells and tendon fibroblasts).

Level of strain	General outcomes
1–4%	Increased *MKX*, *TNMD*, *COL1A1*, *COL1A2* gene expression in tenocytes Kayama et al. (2016)
	Increased total collagen production Riboh et al. (2008), Zhang and Wang, (2010)
	Increased BMP-2 production Rui et al. (2011)
5–8%	Increased *SCX, MKX, TNMD, COL1A2* Wang et al. (2018b)
	Increased *SOX9, RUNX2* in TDSCs but not tenocytes Zhang and Wang, (2010); Zhang and Wang, (2013)
	Increased collagen I, decorin Chen et al. (2007)
	Increased BMP-2 production Rui et al. (2011)
9%+	Increased *TNC* Zhang et al. (2008)
	Increased collagen I, collagen III Zhang et al. (2008)

Moderate-to-high physiologic loads of approximately 4–8% increased ECM and tenogenic gene expression, (Chen et al., 2007; Nam et al., 2019), though some studies also noted a concomitant increase in osteogenic, adipogenic, and chondrogenic markers.(Zhang and Wang, 2013; Nie et al., 2021). This may be due to cell types used in the study. Zhang and Wang found that tendon-derived stem cells stimulated by 8% strain expressed higher levels of non-tendon genes, including transcription factor *SOX-9* and *RUNX2*, while tenocytes did not.(Zhang and Wang, 2013). Upregulation of inflammatory cytokine genes including COX-2 and PGE2 have also been noted at 8% strain by Yang et al., (Yang et al., 2005), suggesting that moderate levels of strain can spur some degree of inflammation and tendon matrix breakdown; conversely, the same study found that 4% strain lowered levels of COX-2, matrix metalloproteinase 1 (MMP-1), and prostaglandin E2 (PGE2).

Studies of effects of higher levels of strain from 9 to 12% begin to demonstrate inflammation and cell death thought to be involved in tendinopathy. Cyclic 10% stretching of mesenchymal stem cells resulted in higher gene expression of collagen I and III and tenascin C after 24 h, (Zhang et al., 2008), but tendon cells also transiently upregulated gene expression of angiogenic factors under 10% biaxial strain, suggesting that the development of tendinopathy may involve angiogenesis driven by tenocyte mechanoresponses. (Mousavizadeh et al., 2013). Higher mechanical loads also increased tenocyte apoptosis, inflammatory markers, and pain modulators commonly seen in tendinopathy.(Arnoczky et al., 2002b; Wang et al., 2003). To highlight the complex interactions between tendon biology, ultrastructure and biomechanical properties, interestingly, it has been noted that fatigue loading of tendon explants which is known to cause immediate changes in collagen organization and molecular denaturation is not associated with immediate changes in local tissue modulus suggesting that despite ultrastructural changes there is no immediate effect on tissue mechanical properties.(Szczesny et al., 2018).

In summary, moderate levels of static mechanical stimulation *and* dynamic load on 2D models may promote tenogenic differentiation of progenitor cells and maintain a tenocyte-like phenotype. Both low and high levels of strain induce inflammatory changes or osteogenic marker upregulation, demonstrating one way tendon may respond to pathological loads with compensatory cellular changes. However, these findings are not consistently reported across all studies, emphasizing the importance of considering other experiment factors. Both the strain level and the duration for which the strain is applied can influence cell behavior. Furthermore, the type of substrate in the extracellular environment can greatly influence study results as the materials composing the substrate may transmit applied forces differently, possibly even attenuating the magnitude of applied forces. As Han et al. have discussed, significant strain attenuation occurs due to the tendon matrix structure, and the direct application of strain to cells on 2D substrates above 4% may be supraphysiologic (Han et al., 2013). The overapplication of strain as a result may explain variable findings in the studies that have explored higher levels of mechanical loading.

### Three-Dimensional (3D) *in vitro* Loading Models

Although 2D models laid the foundation for the study of tendon mechanobiology, the simplicity of the designs limits our understanding of how tendon cells are affected by the surrounding ECM. Compared to 2D models of the same material and growth factors, stem cells seeded in 3D scaffolds more consistently expressed higher levels of tenogenic gene expression, suggesting that the additional cell-matrix interactions play a significant role in guiding cells down a more tenogenic fate (Kuo and Tuan, 2008; James et al., 2011). Wang et al. have also found that the osteogenic and adipogenic differentiation noted in MSCs on a 2D model was inhibited in cells on a 3D scaffold (Wang et al., 2018b). Generally, 3D models contain cells homogenously seeded in a 3D material; scaffolds can be designed such that their architecture promotes cell alignment. Researchers may also apply additional strain by placing the cell-seeded scaffolds in a bioreactor.

### Effects of Differential Loading Parameters on 3D Models

Similar to 2D model studies, cells seeded in 3D scaffolds have responded differently to varying levels of strain. Available loading conditions are summarized in Supplementary Table S3. Effects of loading parameters are complicated by the inherent stiffnesses of different scaffolds, but tenogenic markers have generally been upregulated in stem cells at strains of 2.5–10% (Tables 4, 5).(Kuo and Tuan, 2008; Scott et al., 2011) Patel et al. reported increased gene expression of matrix metalloproteinases when tenocytes seeded in a polyethylene glycol dimethacylate (PEGDM) fiber-gel scaffold were placed under 5% strain (Patel et al., 2017). Interestingly, the same authors later found that scaffold modulus and stiffness played a more significant role in influencing gene expression than strain; cells seeded in softer fiber scaffolds with lower moduli had increased interleukin six and matrix metalloproteinase 3 compared to those in stiffer fibers (Patel et al., 2018). Increased osteogenic gene expression was also seen with extended underloading (3% strain) of tenocytes (Wang et al., 2021). Conversely, underloading 3D models also offered insight into tendon injury healing processes; a “tenostruct” created from a combination of tendon explant, tendon progenitor cells, and bone-derived macrophages in hydrogels revealed increased macrophage recruitment and progenitor cell recruitment to the underloaded explant (Stauber et al., 2021). Future research may further examine the interactions of different cell populations in 3D loading models.

**TABLE 4:** Overview of outcomes of applied mechanical strain to 3D loading studies using human cells (stem cells and tendon fibroblasts).

Level of strain	General outcomes
1–4%	Increased cell alignmentKuo and Tuan, (2008)
	Increased *SCX*, *MKX*, *TNMD*, *TNC*, *COL3A1* gene productionKuo and Tuan, (2008);Wu et al. (2017);Herchenhan et al. (2020)
	Increased type I collagen, type III collagenKuo and Tuan, (2008)
	Decreased type XII collagen, type XIV collagen, elastinKuo and Tuan, (2008)
9%+	Increased *COL1A1, COL3A1, DCN, SCX, TNC* gene expressionGovoni et al. (2017)
	Increased *EGR1, EGR2, FOS, COX-2* gene expressionHerchenhan et al. (2020)

**TABLE 5:** Overview of outcomes of applied mechanical strain to 3D loading studies using animal cells (stem cells and tendon fibroblasts).

Level of strain	General outcomes
1–4%	Increased *TNC*, *SCX*, *COL1A1*, matrix genes (aggrecan, fibronectin, prolyl hydroxylase)Garvin et al. (2003); Scott et al. (2011)
	Increased *RUNX2*, *ALP*, *OCN* gene productionWang et al. (2021)
	Increased type I collagen, type III collagen, type XII collagenBaker et al. (2011)
	Increased heterotropic ossification and decreased biomechanical strengthWang et al. (2021)

### Substrate Material and Architecture of 3D Scaffolds

While the finding that moderate physiologic levels of mechanical loading generally spurs tenogenesis or maintenance is not unique to 3D models, these models have allowed for further study of matrix-cell interactions. Scaffold materials and architecture can significantly affect the amount and type of force applied to cells and are thus a main focus in the design of 3D models. Innovation in this respect of bioreactor design, as a result, is considerably higher than seen in the design of 2D loading experiments. Anisotropic materials such as fibrous scaffolds may transfer force heterogeneously to cells, while isotropic materials like hydrogels transfers force more homogenously. However, there are useful applications for both types of materials, and current mechanobiology research is focused on combining the properties of both anisotropic and isotropic materials to capture the complexity of native tendon environment. The following section summarizes findings from studies using 3D loading models.

### Hydrogel 3D Models

Hydrogels are a popular 3D *in vitro* loading model used in tendon engineering studies. Their ability to transfer mechanical strain uniformly to all cells offers a more controlled environment of study. Hydrogel biomaterials range from collagen I to fibrin and even decellularized plant tissue (Garvin et al., 2003; Feng et al., 2006; Contessi Negrini et al., 2020; Herchenhan et al., 2020). Decellularized tendon samples have also yielded an ECM scaffold suitable for cell culture, possibly allowing for future researchers to conduct studies with a model that closely resembles native tendon in both architecture and composition.(Youngstrom et al., 2013; Youngstrom and Barrett, 2016). Synthetic hydrogels (e.g., polyethylene glycol, methacrylated gelatin) provide higher control of the gels’ physical properties, but fibrous architecture is lacking in these substrates without additional processing.(Hiraki et al., 2021). Seeding in hydrogels induced longitudinal arrangement of cells, increased tenogenic gene expression, and increased ECM production, with and without additional growth factors.(Awad et al., 2000; Farhat et al., 2012; Govoni et al., 2017; Herchenhan et al., 2020). Of note, compared to static loading, cyclic application of mechanical strain was found to upregulate tenogenic gene markers in multipotent stem cells, particularly *SCX*.(Scott et al., 2011). Aspect ratio and cell seeding density were also found to play important roles in guiding cell phenotype, with longer MSC-collagen gels resulting in more organized collagen fiber arrangement and higher seeding densities yielding more aligned cells (Awad et al., 2000; Nirmalanandhan et al., 2007). In addition to cell-mediated matrix remodeling, various methods have been developed to align matrix fibers within hydrogels. One such method includes embedding magnetic particles to provide cells with aligned contact points (Guo and Kaufman, 2007; Hiraki et al., 2021; Xu et al., 2021, 2021).

Further development of 3D printing techniques later allowed for printing of multilayered scaffolds that improve upon the mechanical strength of hydrogels and support greater matrix organization (Rinoldi et al., 2019; Ciardulli et al., 2020). In addition, studies using gels made from extracellular matrix directly derived from tendon and muscle have also offered new insights into the role of tendon-muscle junction proteins in tendon healing. Gaffney et al. found that compared to collagen gels, ECM gels promoted higher expression of paxillin, an intracellular protein linked to focal adhesion turnover; this suggests that collagen gels may lack a component or architecture that affords key cell-matrix interactions involved in tendon healing (Gaffney et al., 2021).

### 3D Fibrous Scaffolds

Advancements in chemical and biological engineering have brought forth the development of fibrous scaffolds, which have led to new 3D loading scaffolds for tendon regeneration. Fibrous scaffolds provide higher levels of matrix alignment and model the shearing forces created by the sliding and stretching of collagen fibers found in the native tendon (Wu et al., 2018). Today, multiple researchers have used electrospinning methods to create sophisticated, tunable knitted structures of nanofibers whose knitted architecture and flexibility closely mimic the environment created by the native extracellular matrix (Sahoo et al., 2010; Baker et al., 2011). Highly aligned biocompatible fibers could be generated from high-speed spinning of polymer solutions, and the resulting scaffolds also possess greater tensile strength than 2D scaffolds of the same polymers (Bosworth et al., 2013). A nanofiber scaffold facilitated proliferation of bone marrow MSCs, upregulated tendon specific gene expression, and increased collagen 1 and tenascin-C production; moreover, a fibrous scaffold generated tendon tissue of higher strength, improved collagen structure, and greater cell density and alignment (Sahoo et al., 2010; Wang et al., 2016; Tu et al., 2020). Schoenenberger et al. found that a fibrous topography alone was sufficient to promote upregulation of genes for inflammatory markers (e.g., interleukin 1 beta (*IL1B*)*,* tumor necrosis factor (*TNF*)) in macrophages and tendon cells, suggesting that nanofiber scaffolds may also be useful in modulating biological responses in tendon healing (Schoenenberger et al., 2020).

To address the nanofiber model’s weak tensile strength, heterogenous fiber architecture, and insufficient mechanical support for clinical translation, later models combined multiple fiber substrates, embedded fibers in a hydrogel, or created microfiber/nanofiber yarns using novel spinning techniques (Liu et al., 2017; Wu et al., 2017; Patel et al., 2018; Lin et al., 2019; Cai et al., 2020). Patel et al. embedded PEGDM fibers in a hydrogel matrix, allowing researchers to replicate the low-shear, high-tension strain experienced by collagen fibers in the native tissue and producing similar findings to collagen based 3D models (Patel et al., 2018). A self-assembling nanofiber hydrogel made of RADA peptides has also been developed and has been shown to promote alignment and growth of tendon progenitor cells (Yin et al., 2020). A polycaprolactone/Poly (l-lactide-co-ε-caprolactone) nanofiber “yarn” developed by Cai et al. promoted tenogenic differentiation of bone marrow MSCs and retained cytocompatibility and biomechanical strength in *in vivo* rabbit studies (Cai et al., 2020). The nanofiber scaffold thus shows promise as a clinically applicable model for tendon tissue engineering. Further study of the effects of dynamic loading conditions applied to electrospun scaffolds may reveal additional findings about the biomechanical properties of tendon.

3D loading models have afforded researchers greater control over the “matrix” architecture in which they are seeding their cells for study. Consistent with 2D models, moderate levels of loading yielded results indicating tenogenic differentiation or tenocyte maintenance, but the sophistication of the scaffold designs offers a deeper understanding of the effects of the elastic moduli of the substrate itself and the additional forces experienced by native tendon, such as shearing or sliding forces.

## Considerations and Future Directions

The study of tendon mechanobiology using mechanical stimulation models has evolved substantially as researchers develop new techniques to more closely model the native tendon environment. The range of loading parameters, cell types, and substrates used in these studies demonstrates the complexity with which researchers must contend. As mechanical loading models grow more sophisticated, researchers must consider several issues when designing studies and translating these approaches from *in vitro* to *in vivo* investigations.

As seen above, studies have employed a wide variety of cell types, substrates, and loading parameters. Variability in loading protocols for both 2D and 3D loading models may make it more difficult for future researchers to reproduce results from loading studies. Certain loading parameters that may drive tenogenic differentiation in one study may not elicit significant changes in another. Moderate levels of strain from 4 to 10% more often yielded tenogenic gene expression, but it was not consistently reported in studies whether other effects, such as upregulation of inflammatory markers or osteogenic genes, increased as well. Differences in results may be attributed to cell types or substrates used in the experiments, though there was no consistent trend in what genes each study tested, making comparison between cell types at the same levels of strain difficult. Responses may even vary by tendon type, which has been shown to have variations on mechanical properties and responses to loading (Zuskov et al., 2020). In addition, many bioreactors used in these studies are custom-designed, and the parameters of the reactor design may also influence cellular response. Future bioreactor studies may explore the unique properties of different tendon types, though it is unclear whether the properties seen in explant studies can be replicated in vitro models. Finally, although certain gene expression levels and protein markers are commonly used to confirm tenogenesis, studies often differ in which markers they analyze. For example, examination of ECM gene expression and protein levels may be a useful but incomplete measure of tendon differentiation, as these genes may also be upregulated in chondrogenesis or osteogenesis.

Experimental designs are also increasing in complexity in order to more closely recapitulate the biomechanical environment of native tendon, but the ability to fully capture the complexity of native biomechanical properties remains limited. Tensile strain has proven sufficient to spur some degree of tenogenic differentiation. However, as recent explant studies have revealed, there are still forces at the fascicular level of tendon structure, such as shear stress or sliding, that have not been deeply examined *in vitro* for their role in governing cellular behavior (Thorpe et al., 2015; Lee and Elliott, 2019). Non-explant *in vitro* models have yet to reach the point where they can be used to study macroscale responses to mechanical strain. While tensile strain has been shown to sufficiently drive tenogenic differentiation or preserve tendon-specific genes in a cell population, further research is required before constructs generated from bioreactors are comparable to the gold standard of autograft and/or allograft tissues used clinically for tendon repair procedures today.

Current research continues to fill in gaps in our understanding of the cellular properties of tendon as well. For example, we are now learning that cellular populations found in tendon may be more diverse and heterogeneous than previously thought, (De Micheli et al., 2020), and lineage tracing has revealed variation in cell populations seen at different severities, stages of tendon injury, and even age may play a significant role in tendon healing as well. (Dyment et al., 2014; Howell et al., 2017; Moser et al., 2018; Nichols et al., 2019). Also noteworthy are interactions within the musculotendinous unit and bone, which may moderate tendon morphology and thus function at the organ level. (O’Neill et al., 2015). *In vitro* studies have yet to incorporate this level of cellular and structural complexity into experimental design, but these may be worthwhile to explore in future research as there remains much to be understood about how the cell-level responses seen in vitro studies may be modulated by higher level interactions. Tendons are ultimately composed of hierarchical structures, and certain mechanical properties may only manifest at higher levels of organization which are not yet achieved through current bioreactor models.

In summary, *in vitro* mechanical loading models of tenogenic cell phenotypes have advanced our understanding of tendon mechanobiology and have equipped investigators with valuable tools to further explore tendon intracellular machinery, its interactions with extracellular matrix and signaling molecules. As discussed in this review, described models are widely varied, offering researchers a diverse range of bioreactors and loading parameters to reference for future research. Each model and loading scheme are associated with unique advantages and disadvantages that can be tailored to address the desired research questions of interest. Moving forward, more sophisticated mechanical loading models that better recapitulate physiologic loading will allow further exploration of the complex interactions between surrounding tissues (bone, muscle, neurovasculature, etc.) and tendon itself. Ultimately, this may deepen our understanding of the relevant molecular mechanisms associated with tendon development and pathogenesis and may provide the foundation for future tendon tissue healing and regeneration approaches.
