# Supplementary material for: In Vitro Cellular Strain Models of Tendon Biology and Tenogenic Differentiation
Source: Front Bioeng Biotechnol. 2022 Feb 15;10:826748. doi: 10.3389/fbioe.2022.826748 (PMC8886160; doi:10.3389/fbioe.2022.826748)
Supplement: Supplementary file 1 [file DataSheet1.docx]

Supplementary Material

# Explant dynamic loading conditions reported in the literature

**Table 1.** Summary of explant models using dynamic mechanical loading. Loading strain (documented as percent surface elongation), loading parameters (frequency of loading and duration), cell type, and study findings. Strain is uniaxial unless stated otherwise. Durations of strain under 24 hours are given as number of hours per day unless stated otherwise. Unless stated otherwise, study findings are summarized for the strain and loading parameters documented.

| **Strain** | **Loading parameters** | **Explant type** | **Findings** | **Source** |
| --- | --- | --- | --- | --- |
| 3 or 12 MPa, strains up to 2% | 1 or 12 days | Avian flexor digitorum profundus | Loading to 12MPa reduced maximum stress and energy density  3MPa caused reduction in energy density when applied for 12 days  Cell death, collagenase activity increased with increasing magnitude and time; no differences in cell viability or collagen content  GAG content increased, PGE2 increased with loading magnitude and duration | (Devkota et al., 2007) |
| 30-60% | 1Hz, 15min-5h | Bovine extensor tendon fascicles | Increased *COL1A1, IL-6* | (Legerlotz et al., 2013) |
| Low, medium, high magnitude loading corresponding to force-displacement curve (10-75% of ultimate tensile strength) | 1Hz, 10 or 1000 cycles | Mouse patellar tendons, injured vs. uninjured | Decreased strain stiffening and dynamic modulus in highly strained samples  Increased collagen disorganization and decreased strain transfer to nuclei in fatigue loaded samples | (Freedman et al., 2018) |
| 1% cyclic | 1Hz, 8h | Mouse tendon fascicles | Tendon-like morphology preserved in explants under strain with concomitant preservation of gene expression of tendon specific and matrix remodeling genes of native tissue, e.g., *SCX, TNMD, Ctgf, MMP3, MMP9* | (Wunderli et al., 2018) |
| 3%, 6%, 9% | 0.25Hz, 8h/day | Rabbit Achilles tendons | Moderate matrix deterioration and elevated collagenase levels at 3% strain  Retained structural integrity and cellular function at 6% strain  Massive collagen bundle rupture at 9% | (Wang et al., 2013) |
| Load-to-failure |  | Rat flexor carpi ulnaris tendons | Increased collagen fiber kinking and collagen denaturation with no changes in tissue modulus | (Szczesny et al., 2018) |
| 2.5% | 1Hz, 6,24,48h | Rat flexor digitorum longus tendons | Heightened *scx* expression after 6, 24h of stimulation, declined at 48h.  Increased MMP-1 and MMP-13 protein  Increase in mechanical strength of tendon during first half of loading time, decreased strength in latter half | (Tohidnezhad et al., 2020) |
| Plantaris: 14%, 20%, 25%  Tail: 2%, 4%, 6%, 8% |  | Rat plantaris and tail tendons | Attenuation of applied strain at the micro-scale level, facilitated by forces such as shearing | (Lee and Elliott, 2019) |
| 5% strain | 1Hz | Rat tail tendon fascicles | Upregulation of collagen synthesis over 24 h | (Screen et al., 2005) |
| 3% strain imposed on 2% static strain | 10 min | Rat tail tendon fascicles | 6h of strain significantly increased type III collagen expression  Downregulation of *decorin at 24h*  increased *MMP3, MMP13* with 10 min strain  catabolic genes were upregulated by small number of strain cycles, downregulated by prolonged cyclic strain | (Maeda et al., 2009) |
| 0 – 10% strain | rates: 0.01%/s; 0.1%/s; 1.0%/s | Rat tail tendon fascicles | Sensing of shear stresses by ion channel PIEZO1, as demonstrated by Ca2+ imaging:  At low strain, tenocytes expressed multiple Ca2+ signals. At high strain, tenocytes expressed single Ca2+ signal, with higher tissue stretch required to elicit response in 50% of cells. | (Passini et al., 2021) |
| 20% strain | 1Hz, 6hr | Rat tibialis anterior tendons | Induced stretch overload injury with increased apoptosis, abnormal nuclear morphology (including fragmentation) | (Scott et al., 2005) |

# 2D in vitro dynamic loading conditions reported in the literature

**Table 2.** Summary of 2D models using dynamic mechanical loading. Loading strain (documented as percent surface elongation), loading parameters (frequency of loading and duration), cell type, and study findings. Strain is uniaxial unless stated otherwise. Durations of strain under 24 hours are given as number of hours per day. Unless stated otherwise, study findings are summarized for the strain and loading parameters documented.

| **Strain** | **Loading parameters** | **Cell types investigated** | **Findings** | **Source** |
| --- | --- | --- | --- | --- |
| 3%, 10% | 1Hz, 8 or 48 hrs | Human BMSCs | Increased mRNA of type I collagen, type III collagen, *TNC* in 10%, 48hr group  Transient increase of *ALP* at 8hr, returned to baseline by 48hr in 3% group | (Chen et al., 2008) |
| 4%, 8%, 12% | 1Hz, 6-72 hrs | Human BMSCs | Increased collagen I, collagen III, fibronectin, N-cadherin in 8%, 12% groups  Increased *SCX, TNMD, TNC* in 8%, 12% groups  Transiently increased *RUNX2, ALP, OCN* in 4% group | (Nam et al., 2019) |
| 4%, 8% | 0.5Hz, 4 hrs | Human patellar tendon fibroblasts | Increased cell proliferation at 4% and 8%, significant in 8%  Increased collagen type I, TGF-β at 4% and 8%, higher in 8% | (Yang et al., 2004) |
| 4%, 8% | 0.5Hz, 4 hrs | Human patellar tendon fibroblasts | Decreased COX-2, MMP-1 gene expression, PGE2 production in 4% group  Increased COX-2, MMP-1 gene expression, PGE2 production in 8% group | (Yang et al., 2005) |
| 5% | 1Hz, 15 or 60 min | Human tendon fibroblasts | Increased JNK activation (highest in 15 min)  Increased apoptosis (15 min) | (Skutek et al., 2003) |
| 4%, 8%, 12% | 0.5Hz, 24 hrs | Human tendon fibroblasts | Increased PGE2 in 8% and 12% groups  Increased COX-1, COX-2 for all stretching groups, with exception of COX-1 in 4% | (Wang et al., 2003) |
| 10% biaxial* 1Hz | 1Hz | Primary human tenocytes | Transiently increased *ANGPTL4,* FGF-2*, COX-2, SPHK1*, TGF-alpha*, VEGF-A* and *VEGF-C* | (Mousavizadeh et al., 2014) |
| 3.5% | 1Hz, 2 hrs | Human tenocytes | Increased IL-1β, COX-2, MMP-3 gene expression | (Tsuzaki et al., 2003) |
| 6% biaxial vs. uniaxial | 0.25Hz, 8 hrs | Murine TDSCs | Increased *SCX*, *MKX*, *TNMD*, *COL1A2* in uniaxial group | (Wang et al., 2018) |
| 8% | 12 hrs | Murine TDSCs, tenocytes | Increased tenocyte and non-tenocyte genes, including *SOX9, RUNX2*, in TDSCs but not tenocytes | (Zhang and Wang, 2013) |
| 10% | 1Hz, 3-36 hrs | Rat BMSCs | Increased Collagen I, Collagen III after 12 hrs  Increased *TNC* after 24 hrs | (Zhang et al., 2008) |
| 4%, 8% | 0.5Hz, 4 hrs | Rat TDSCs | Increased expression of BMP-2 protein at both 4% and 8% stretching groups | (Rui et al., 2011) |
| 2% | 0.25Hz, 6h | Rat tenocytes | Increased *MKX, TNMD, COL1A1, COL1A2* | (Kayama et al., 2016) |
| 5% | 0.5Hz, 24 hrs | Porcine patellar tendon fibroblasts | Increased type I collagen, decorin | (Chen et al., 2007) |
| 4% | 0.1 Hz, 1 hr on/2-5 hours off | Rabbit ASCs, tendon sheath fibroblasts, BMSCs, epitenon tenocytes | Increased total collagen production in ASCs, sheath fibroblasts  Increased cell proliferation of sheath fibroblasts | (Riboh et al., 2008) |
| 4%, 8% | 0.5Hz, 12 hrs | Rabbit TDSCs | Increased collagen I in 4%, 8% groups  Increased *SOX9*, *RUNX2* in 8% group | (Zhang and Wang, 2010) |

# 3D in vitro tendon dynamic loading conditions reported in the literature

**Table 3**. Summary of 3D scaffolds using dynamic mechanical loading. Inherent mechanical properties of scaffolds, such as modulus or fluid shear, are not included. Loading strain (typically documented as percent surface elongation), loading parameters (frequency of loading and duration), cell type, and study findings. Durations of strain under 24 hours are given as number of hours per day. Unless stated otherwise, study findings are summarized for the strain and loading parameters documented.

| **Strain** | **Loading parameters** | **Cell types** | **Findings** | **Source** |
| --- | --- | --- | --- | --- |
| 1% | 1Hz, 30 min | Human BMSCs | Increased cell alignment  Increased *SCX*, Collagen type III  Decreased Collagen types XII, XIV, elastin | (Kuo and Tuan, 2008) |
| ~10% (0.1N force) | 1Hz | Human BMSCs | Increased *COL1A1, ColL3A1, DCN, SCX, TNC* in presence of both strain and hGDF-5 after 3 days | (Govoni et al., 2017) |
| 0.09 Pa | 4 hrs | Human BMSCs | Increased Collagen, Scleraxis, and Decorin  Increased “pro-inflammatory (*IL-6, TNF, IL-12A, IL-1β*) and anti-inflammatory (*IL-10, TGF-β1*) cytokine gene expressions, with a significant increase of anti-inflammatory cytokines in dynamic conditions.” | (Ciardulli et al., 2020) |
| 4% | 0.5hz, 2 hrs | Human primary ASCs | Increased *SCX*, Collagen I, and *TNMD* gene expression | (Wu et al., 2017) |
| 2.5%, 10% | 1Hz, 5 min | Human tendon fibroblasts | Increased *EGR2, MKX, TNMD, COL3A1* in 3D constructs, without strain  Increased *EGR1, EGR2, FOS, COX-2* at 10% strain | (Herchenhan et al., 2020) |
| 2.5%, 5%, 7.5%, 10% | 0.1Hz, 2 hrs | Murine MSCs, tenocytes | “Cyclic loading resulted in a greater increase of tenocyte gene expression than static loading over 3 weeks. Increasing strain levels potentiated the induction of tenocyte genes [*SCX* and *COL1A1*]. The insertion of a 10 s rest periods further enhanced tenocyte gene expression, as did increasing repetition numbers.” | (Scott et al., 2011) |
| 3% | 0.25Hz, 8 hrs | Murine TDSCs | Progressive heterotropic ossification and decreased biomechanical strength  Increased *RUNX2, ALP, OCN* in 3% strain group | (Wang et al., 2021) |
| 1% | 1Hz, 1 hr | Avian tendon fibroblasts | Increased collagen I, collagen III, collagen XII, *TNC*, matrix genes (aggrecan, fibronectin, prolyl hydroxylase) in 3D constructs comparable to 2D culture | (Garvin et al., 2003) |
| 3% | 1Hz | Bovine MSCs | Increased collagen I, fibronectin | (Baker et al., 2011) |

**Definitions:**

***ALP*:** Alkaline phosphatase; ***ANGPTL4***: Angiopoietin Like 4; **ASCs:** Adipose-derived stem cells; ***BMP-2*:** bone morphogenic protein 2; **BMSCs**: bone marrow derived stem cells; ***COL1A1*:** collagen 1, alpha-1; ***COL1A2*:** collagen 1, alpha-2; ***COL3A1*:** collagen 3, alpha-1; **COX:** cyclooxygenase; ***Ctgf*:** connective tissue growth factor; ***DCN*:** decorin; ***EGR2*:** early growth response 2; **FGF-2**: fibroblast growth factor 2; **hGDF-5:** human growth differentiation factor 5; **IL:** interleukin; **JNK:** Jun N-terminal kinase; ***MKX*:** Mohawk; **MMP-1**: matrix metalloproteinase 1; ***OCN*:** Osteocalcin; **PGE2:** prostaglandin E2; ***RUNX2*:** Runt-related transcription factor 2; ***SCX*:** Scleraxis; ***SPHK1*:** sphingosine kinase 1; **TDSCS:** tendon-derived stem cells; **TGF- β:** transforming growth factor beta; **TGF-α:** transforming growth factor alpha; ***TNC*:** Tenascin-C; ***TNF*:** tumor necrosis factor; ***TNMD*:** Tenomodulin; **VEGF:** vascular endothelial growth factor
